# Interobserver agreement and prognostic impact for MRI–based 2018 FIGO staging parameters in uterine cervical cancer

**DOI:** 10.1007/s00330-022-08666-x

**Published:** 2022-03-24

**Authors:** Kari S. Wagner-Larsen, Njål Lura, Øyvind Salvesen, Mari Kyllesø Halle, David Forsse, Jone Trovik, Noeska Smit, Camilla Krakstad, Ingfrid S. Haldorsen

**Affiliations:** 1 Department of Radiology, Mohn Medical Imaging and Visualization Centre MMIV, Haukeland University Hospital, Jonas Lies vei 65, N-5021 Bergen, Norway; 2 Section for Radiology, Department of Clinical Medicine, University of Bergen, Bergen, Norway; 3 Clinical Research Unit, Department of Clinical and Molecular Medicine, Norwegian University of Science and Technology, Trondheim, Norway; 4 Department of Obstetrics and Gynecology, Haukeland University Hospital, Bergen, Norway; 5 Centre for Cancer Biomarkers CCBIO, Department of Clinical Science, University of Bergen, Bergen, Norway; 6 Department of Informatics, University of Bergen, Bergen, Norway

**Keywords:** Uterine cervical neoplasms, Magnetic resonance imaging, Observer variation, Prognosis, Risk assessment

## Abstract

**Objectives:**

To evaluate the interobserver agreement for MRI–based 2018 International Federation of Gynecology and Obstetrics (FIGO) staging parameters in patients with cervical cancer and assess the prognostic value of these MRI parameters in relation to other clinicopathological markers.

**Methods:**

This retrospective study included 416 women with histologically confirmed cervical cancer who underwent pretreatment pelvic MRI from May 2002 to December 2017. Three radiologists independently recorded MRI–derived staging parameters incorporated in the 2018 FIGO staging system. Kappa coefficients (*κ*) for interobserver agreement were calculated. The predictive and prognostic values of the MRI parameters were explored using ROC analyses and Kaplan–Meier with log-rank tests, and analyzed in relation to clinicopathological patient characteristics.

**Results:**

Overall agreement was substantial for the staging parameters: tumor size > 2 cm (*κ* = 0.80), tumor size > 4 cm (*κ* = 0.76), tumor size categories (≤ 2 cm; > 2 and ≤ 4 cm; > 4 cm) (*κ* = 0.78), parametrial invasion (*κ* = 0.63), vaginal invasion (*κ* = 0.61), and enlarged lymph nodes (*κ* = 0.63). Higher MRI–derived tumor size category (≤ 2 cm; > 2 and ≤ 4 cm; > 4 cm) was associated with a stepwise reduction in survival (*p* ≤ 0.001 for all). Tumor size > 4 cm and parametrial invasion at MRI were associated with aggressive clinicopathological features, and the incorporation of these MRI–based staging parameters improved risk stratification when compared to corresponding clinical assessments alone.

**Conclusion:**

The interobserver agreement for central MRI–derived 2018 FIGO staging parameters was substantial. MRI improved the identification of patients with aggressive clinicopathological features and poor survival, demonstrating the potential impact of MRI enabling better prognostication and treatment tailoring in cervical cancer.

**Key Points:**

*• The overall interobserver agreement was substantial (κ values 0.61–0.80) for central MRI staging parameters in the 2018 FIGO system.*

*• Higher MRI–derived tumor size category was linked to a stepwise reduction in survival (p ≤ 0.001 for all).*

*• MRI–derived tumor size > 4 cm and parametrial invasion were associated with aggressive clinicopathological features, and the incorporation of these MRI–derived staging parameters improved risk stratification when compared to clinical assessments alone.*

**Supplementary Information:**

The online version contains supplementary material available at 10.1007/s00330-022-08666-x.

## Introduction

Uterine cervical cancer is the fourth most common cancer among women worldwide, and one of the leading causes of cancer-related deaths, especially in low- and middle-income countries [1]. Cervical cancer is staged according to the International Federation of Gynecology and Obstetrics (FIGO) system [2]. The previous 2009 FIGO classification was primarily based on clinical examinations with limited incorporation of information from additional diagnostic procedures [3]. Thus, cross-sectional imaging findings, though commonly used to guide treatment decisions in high-resource settings, were not included in the staging [4, 5]. Recognizing this disparity, the recently revised 2018 FIGO system formally incorporates results from available diagnostic imaging and pathology assessments into stage assignment [2]. 2018 FIGO subdivides stage IB into IB1–3 based on tumor size, and assigns lymph node metastases to stage IIIC. Better risk stratification between 2018 FIGO stages than between 2009 FIGO stages has been reported [6–8], and large tumor size, parametrial invasion, and nodal involvement are uniformly reported to predict poor outcome in cervical cancer [6–13].

Pelvic magnetic resonance imaging (MRI) is the imaging modality of choice for local and regional staging of macroscopically visible cervical cancer at primary diagnostic work-up [14, 15]. The superiority of MRI over clinical examination for accurate assessments of tumor size, parametrial invasion, and vaginal extension is well documented [16–20]. Knowledge of interobserver reproducibility for MRI–based 2018 FIGO staging parameters is, however, a key element in establishing the validity of MRI. Previous MRI studies report variable interobserver agreement for the central staging parameters: tumor size categories (*κ* = 0.46) [21], parametrial invasion (*κ* = 0.45–0.90) [19, 22–24], vaginal invasion (*κ* = 0.36/0.47) [23], and pelvic/paraaortic lymph node metastases (*κ* = 0.45–0.81) [19, 23]. Furthermore, the literature is scarce on how these MRI–derived staging parameters are linked to other clinicopathological markers and how they may aid in prognostication.

This study aimed to evaluate the interobserver agreement for MRI–based 2018 FIGO staging parameters at pretreatment MRI in a large cervical cancer patient cohort, and assess the potential prognostic value of these MRI parameters in relation to clinical 2009 FIGO stage and clinicopathological markers.

## Materials and methods

### Patients and study setting

This retrospective study on prospectively collected data was approved by the Regional Committee for Medical Research Ethics (2015/2333/REK vest) with written informed consent at primary diagnosis from all patients.

From May 2002 to December 2017, pelvic MRI was performed as part of clinical routine at primary diagnostic work-up in 420 women with histologically confirmed cervical cancer. Four patients had incomplete MRI (*n* = 2) or missing follow-up data (*n* = 2), leaving 416 patients eligible for study inclusion. All patients were diagnosed and treated at Haukeland University Hospital. Clinical data (e.g., clinical tumor size and 2009 FIGO stage) were registered. Patients originally staged according to the 1994 FIGO system were later restaged based on the 2009 FIGO staging criteria. Histopathological variables and follow-up data were collected from the medical records. Progression was defined as local recurrence/progression in the pelvis or new metastases in the abdomen or at distant sites, confirmed by clinical examination with biopsy, or by imaging (computed tomography (CT), MRI, and/or ^18^F-fluorodeoxyglucose positron emission tomography with CT (FDG-PET/CT)). Patients presenting with new imaging findings regarded as highly likely to represent progression (e.g., growth of known tumor mass or new lesions/new FDG-PET positive lesions in patients without previous history of other malignancies as potential origins of metastases) were categorized as recurrence (even without histological verification). Imaging findings regarded as unsure or possible (but not indicative of) progression, in patients without a positive biopsy, were categorized as no recurrence. Date of last follow-up was September 2021. During the follow-up, 89 patients experienced progression with a median (mean) [interquartile range, IQR] time to progression of 11 (16) [7–24] months. Median (mean) [IQR] follow-up for survivors was 91 (101) [65–127] months.

### MRI protocol

Pelvic MRI was acquired on scanners from different manufacturers (GE Healthcare, Siemens Healthineers, Philips Healthcare), comprising 1.5-T (329/416 patients) or 3.0-T (87/416 patients) systems, at five hospitals in Western Norway. The imaging protocols and scanning parameters varied across scanners and institutions, reflecting current guidelines and local preferences. All examinations were, however, dedicated pelvic protocols largely in accordance with European Society of Urogenital Radiology (ESUR) guidelines for MRI staging of cervical cancer [14]. As a minimum, the protocols included axial and/or axial oblique (perpendicular to the long axis of the uterine cervix), sagittal, and coronal and/or coronal oblique (parallel to the long axis of the cervix) T2-weighted (T2W) sequences in addition to an axial T1-weighted (T1W) sequence of the pelvis. In total, 66% (273/416) of the examinations included a pelvic diffusion-weighted imaging (DWI) sequence. During the study period, contrast-enhanced T1W series were not routinely included in the MRI protocols and thus were only performed in 10% (40/416) of the patients. A detailed overview of MRI acquisition parameters in a subset of the patients (*n* = 123) is given in Suppl. Table 1.

### Image analysis

**Fig. 1:**
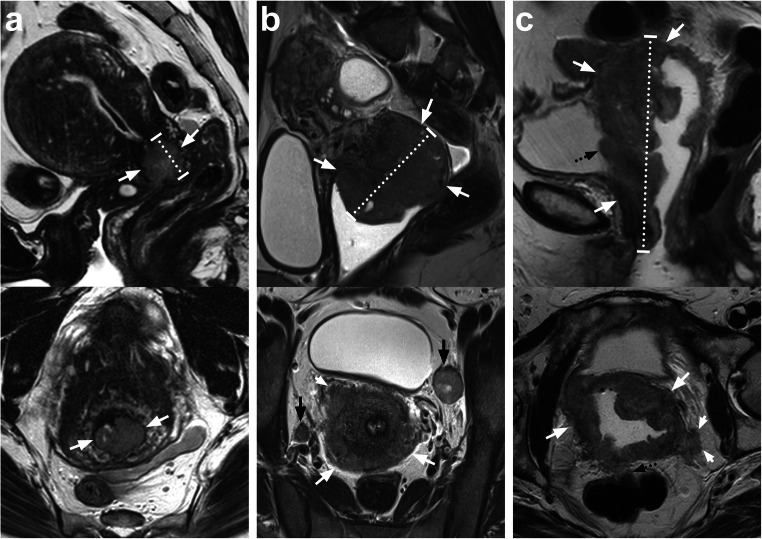
Cervical cancer depicted by sagittal (top) and axial oblique (bottom) T2-weighted MRI views in three patients. **a** A 40-year-old woman with a moderately large cervical cancer (white arrows) with a maximum tumor size of 2.4 cm (dotted line). The tumor is confined to the cervical stroma, and there are no enlarged lymph nodes (2018 FIGO IB2). The patient received primary surgical treatment (radical hysterectomy and salpingectomy) and had no signs of recurrence at 4 years post treatment. **b** A 23-year-old woman with a large cervical cancer (white arrows) with a maximum tumor size of 6.0 cm (dotted line). The tumor invades the parametrium (short white arrow), and bilateral enlarged pelvic lymph nodes are depicted (black arrows) (2018 FIGO IIIC1). The patient was treated with primary chemoradiation therapy and died from cervical cancer 2.5 years after primary diagnosis. **c** A 70-year-old woman with a large, irregular cervical cancer (white arrows) that extends to the uterine fundus and the lower third of the vagina. The maximum tumor size is 10.0 cm (dotted line) and tumor invades the parametrium (short white arrows) and both the bladder and rectum (black dotted arrows) (2018 FIGO IVA). The patient received primary chemoradiation therapy and died from cervical cancer 8 months after primary diagnosis. FIGO, International Federation of Gynecology and Obstetrics

The MRI examinations were de-identified and reviewed independently by three radiologists blinded to clinical and histopathologic information. Reader 1 (N.L.), 2 (K.W.L.), and 3 (I.J.M.) were consultants from the same institution with 5, 10, and 20 years of experience, respectively, with pelvic MRI. The readers reported MRI findings relevant for 2018 FIGO staging [25] in a standardized form including both continuous and categorical variables. Maximum tumor diameters and depth of parametrial invasion were measured regardless of plane on T2W images (Fig. 1) and later categorized (≤ 2 cm; > 2 and ≤ 4 cm; > 4 cm, absence/presence of parametrial invasion). Patients with no visible tumor were recorded with maximum tumor size ≤ 2 cm. Regardless of tumor visibility on MRI, all images were analyzed systematically for relevant findings (e.g., enlarged lymph nodes). Imaging findings suggesting vaginal invasion (upper two-thirds or lower third), bladder-/rectum- or pelvic-sidewall invasion, hydroureter (indicative of hydronephrosis), and enlarged pelvic/paraaortic lymph nodes suspicious of metastases were assessed on T2W images, supported by T1W and DWI sequences when available (Fig. 1). Diagnostic criteria of parametrial invasion were full-thickness cervical stroma invasion co-occurring with spiculated or nodular tumor-to-parametrium interface and/or encasement of parametrial vessels. Vaginal invasion was defined as tumor disruption of the vaginal wall, and bladder/rectum involvement was diagnosed when the bladder or rectal wall was interrupted with tumor nodules in the mucosa. Pelvic-sidewall invasion was defined as tumor extending into the iliac vessels, internal obturator, piriformis, or levator ani muscles. Pelvic/paraaortic lymph nodes were considered suspicious of metastases if they had  > 1 cm short-axis diameter [26].

To establish the overall imaging findings based on the recordings by all three readers, “consensus reading” variables were generated using the median values recorded for the continuous variables and the category recorded by the majority for the dichotomous variables.

To ensure a common understanding of the image reading criteria applied, the readers and an expert in gynecologic cancer imaging (I.S.H.) independently filled in the registration form for five randomly selected pilot cases prior to the review of the entire patient cohort. Disagreements in interpretation were discussed to reach a consensus.

### Statistical analysis

Pairwise and overall interobserver agreement was assessed using Cohen’s, Fleiss’, and weighted kappa (*κ*) statistics. Agreement beyond chance was interpreted as slight (*κ* ≤ 0.20), fair (*κ* = 0.21–0.40), moderate (*κ* = 0.41–0.60), substantial (*κ* = 0.61–0.80), and almost perfect (*κ* > 0.81) [27].

To compare the diagnostic performance of the different imaging parameters for prediction of disease-specific death at 5 years after primary diagnosis, time-dependent receiver operating characteristic (ROC) analyses were used. The prognostic value of the different imaging parameters was explored using the Cox proportional hazard model and Kaplan–Meier with log-rank tests. Chi-square test was used to analyze the imaging parameters in relation to clinicopathological patient characteristics. Test of equal area under the ROC curves (AUC) among the three readers and the consensus reading, and among the different MRI–derived staging parameters (consensus reading), was performed using 6 and 15 pairwise comparisons of AUCs, respectively. *p* values were adjusted according to the Holm–Bonferroni method, yielding significance levels less than 0.008 (0.05/6) and 0.005 (0.05/10), respectively. All other *p* values were considered significant when less than 0.05 (two-sided). The data were analyzed using R 4.0.3 (TimeROC package [28], R Core Team 2020 [29]), SPSS 26.0 (IBM Corp.), and STATA 16.1 (StataCorp).

## Results

### Patient characteristics and primary treatment

**Fig. 2:**
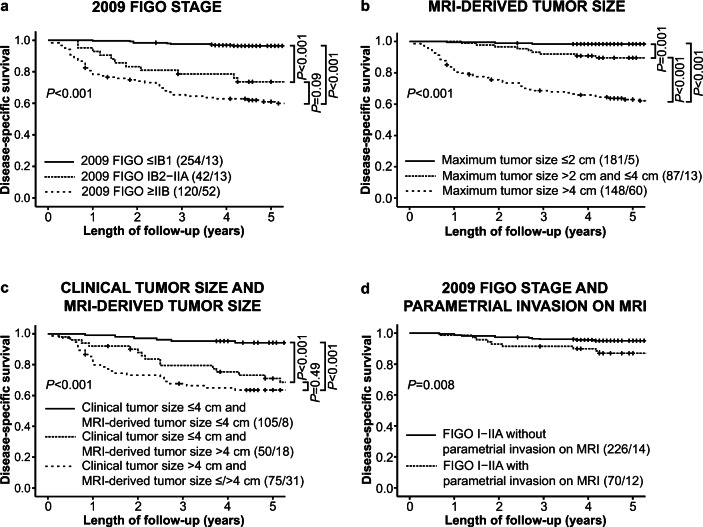
Kaplan–Meier survival curves depicting significantly reduced disease-specific survival in patients with (**a**) 2009 FIGO stages IB2–IIA and ≥ IIB compared to stages ≤ IB1, (**b**) higher MRI–derived tumor size categories, (**c**) clinical tumor size ≤ 4 cm but MRI–derived tumor size > 4 cm, (**d**) 2009 FIGO stages I–IIA but parametrial invasion at MRI. For each category: total number of cases/number of cases with disease-specific death. FIGO, International Federation of Gynecology and Obstetrics

Median age at primary diagnosis in the patient cohort (*n* = 416) was 43 years (IQR 36–55). Altogether, 68% (282/416) of the patients were diagnosed with 2009 FIGO stage I, 19% (80/416) with stage II, 9% (37/416) with stage III, and 4% (17/416) with stage IV (Table 1). Primary treatment consisted of surgery only in 51% (210/416), surgery combined with adjuvant treatment in 12% (51/416), and definitive radiotherapy/chemoradiation in 35% (147/416), whereas 2% (8/416) received palliative treatment (Suppl. Table 2). At last follow-up, 19% (78/416) of the patients had died from the disease. Patients with 2009 FIGO stages IB2–IIA (*n* = 42) and ≥ IIB (*n* = 120) exhibited reduced disease-specific and progression-free survival compared to stages ≤ IB1 (*n* = 254) (*p* < 0.001 for both) (Fig. 2a and Suppl. Figure 1a).

**Table 1:** Clinicopathological characteristics of 416 patients with cervical cancer

	Median	(Interquartile range)
Age, years (*n* = 416)	43	(36–55)
BMI, kg/m^2^ (*n* = 415)	25	(22–28)
	*n*	(%)
Menopausal status (*n* = 409)		
Pre-/perimenopausal	278	(68)
Postmenopausal	131	(32)
Parity (*n* = 416)		
Para 0	59	(14)
Para 1 +	357	(86)
Clinical tumor size (*n* = 230)		
< 2 cm	46	(20)
≥ 2 cm	184	(80)
> 4 cm	75	(33)
2009 FIGO stage (*n* = 416)		
I^a^	282	(68)
II^b^	80	(19)
III^c^	37	(9)
IV^d^	17	(4)
Histologic type (*n* = 416)		
Squamous cell carcinoma	292	(70)
Adenocarcinoma	92	(22)
Other^e^	32	(8)
Histologic grade (*n* = 343)		
1 & 2	253	(74)
3	90	(26)
Primary treatment (*n* = 416)		
Surgery alone^f^	210	(51)
Surgery and adjuvant therapy^g^	51	(12)
Radiotherapy ± chemotherapy alone	147	(35)
Palliative treatment	8	(2)
Status at last follow-up (*n* = 416)		
Alive, without evidence of cervical cancer	313	(75)
Alive, with known cervical cancer	5	(1)
Death from cervical cancer	78	(19)
Death from uncertain or other causes	20	(5)

*BMI* body mass index, *FIGO* International Federation of Gynecology and Obstetrics

^a^Tumor confined to the cervix

^b^Tumor extending beyond the uterus, but not onto the pelvic sidewall or to the lower third of the vagina

^c^Tumor extending onto the pelvic sidewall or to the lower third of the vagina/causing hydronephrosis

^d^Tumor extending beyond the true pelvis or invading bladder and/or rectum

^e^Adenosquamous, neuroendocrine, and undifferentiated carcinomas

^f^Conization, trachelectomy, or hysterectomy ± bilateral salpingectomy/salpingo-oophorectomy

^g^Chemoradiation combined, chemotherapy only, or radiotherapy only

### MRI–derived 2018 FIGO staging parameters at primary diagnostic work-up

In total, 65% (270/416; based on consensus reading) of the patients had visible cervical cancer on MRI (Table 2). These tumors had a median (mean) [IQR] maximum diameter of 43 (45) [30–56] mm. Prevalence of MRI staging parameters for the entire patient cohort is given in Table 2, with corresponding figures for the subgroups of patients with visible tumor (*n* = 276 [reader 1]; *n* = 273 [reader 2]; *n* = 259 [reader 3]; *n* = 270 [consensus reading]) in Suppl. Table 3. The patients with positive MRI findings almost uniformly had visible tumor on the cervix; however, enlarged lymph nodes were recorded in two patients (consensus reading) who did not have visible tumor.

**Table 2:** Prevalence of positive MRI staging parameters (2018 FIGO staging system) for the three readers and the consensus reading at primary diagnostic work-up in 416 patients with cervical cancer

	Reader 1 *n* (%)	Reader 2 *n* (%)	Reader 3 *n* (%)	Consensus reading *n* (%)
Visible tumor	276 (66)	273 (66)	259 (62)	270 (65)
Tumor size > 2 cm	235 (57)	234 (56)	242 (58)	235 (56)
Tumor size > 4 cm	149 (36)	142 (34)	166 (40)	148 (36)
Tumor size, three categories				
≤ 2 cm	181 (44)	182 (44)	174 (42)	181 (44)
> 2 and ≤ 4 cm	86 (21)	92 (22)	76 (18)	87 (21)
> 4 cm	149 (36)	142 (34)	166 (40)	148 (36)
Parametrial invasion	180 (43)	144 (35)	230 (55)	180 (43)
Vaginal invasion	161 (39)	186 (45)	170 (41)	173 (42)
Limited to upper two-thirds	134 (32)	176 (42)	111 (27)	153 (37)
Extension to lower one-third	27 (7)	10 (2)	59 (14)	20 (5)
Pelvic-sidewall invasion	1 (0)	10 (2)	5 (1)	3 (1)
Hydroureter	5 (1)	6 (1)	1 (0)	3 (1)
Enlarged lymph nodes^a^	63 (15)	62 (15)	44 (11)	59 (14)
Bladder/rectum invasion	45 (11)	41 (10)	61 (15)	36 (9)

*FIGO* International Federation of Gynecology and Obstetrics

^a^Defined as pelvic/paraaortic lymph nodes with short-axis diameter > 1 cm

### Interobserver agreement for MRI–derived 2018 FIGO staging parameters

Overall [pairwise] agreement between readers was substantial for tumor size > 2 cm (*κ* = 0.80 [0.75–0.86]), tumor size > 4 cm (*κ* = 0.76 [0.71–0.83]), tumor size categories (≤ 2 cm; > 2 and ≤ 4 cm; > 4 cm) (*κ* = 0.78 [0.73–0.84]), parametrial invasion (*κ* = 0.63 [0.54–0.73]), vaginal invasion (*κ* = 0.61 [0.55–0.68]), and enlarged lymph nodes suggestive of metastases (*κ* = 0.63 [0.51–0.75]) (Table 3). For the remaining staging parameters, agreement was only moderate or fair.

**Table 3:** *κ* values for pairwise and overall interobserver agreement for the evaluation of MRI staging parameters (included in the 2018 FIGO staging system) at primary diagnostic work-up in 416 patients with cervical cancer

	Reader 1–2	Reader 1–3	Reader 2–3	Overall *κ* (95% CI)
Tumor size > 2 cm	0.86	0.78	0.75	0.80 (0.74–0.85)
Tumor size > 4 cm	0.83	0.73	0.71	0.76 (0.70–0.81)
Tumor size, three categories	0.84^a^	0.75^a^	0.73^a^	0.78 (0.74–0.81)^a^
≤ 2 cm				
> 2 and ≤ 4 cm				
> 4 cm				
Parametrial invasion	0.73	0.65	0.54	0.63 (0.58–0.69)
Vaginal invasion	0.68	0.55	0.61	0.61 (0.56–0.67)
Limited to upper two-thirds	0.58	0.33	0.39	0.43 (0.38–0.49)
Extension to lower one-third	0.41	0.31	0.20	0.28 (0.22–0.33)
Pelvic-sidewall invasion	0.18	0.33	0.39	0.30 (0.25–0.36)
Hydroureter	0.54	0.33	0.28	0.41 (0.36–0.47)
Enlarged lymph nodes^b^	0.75	0.63	0.51	0.63 (0.58–0.69)
Bladder/rectum invasion	0.51	0.46	0.49	0.48 (0.43–0.54)

*FIGO* International Federation of Gynecology and Obstetrics, *CI* confidence interval

^a^Weighted kappa

^b^Defined as pelvic/paraaortic lymph nodes with short-axis diameter > 1 cm

**Fig. 3:**
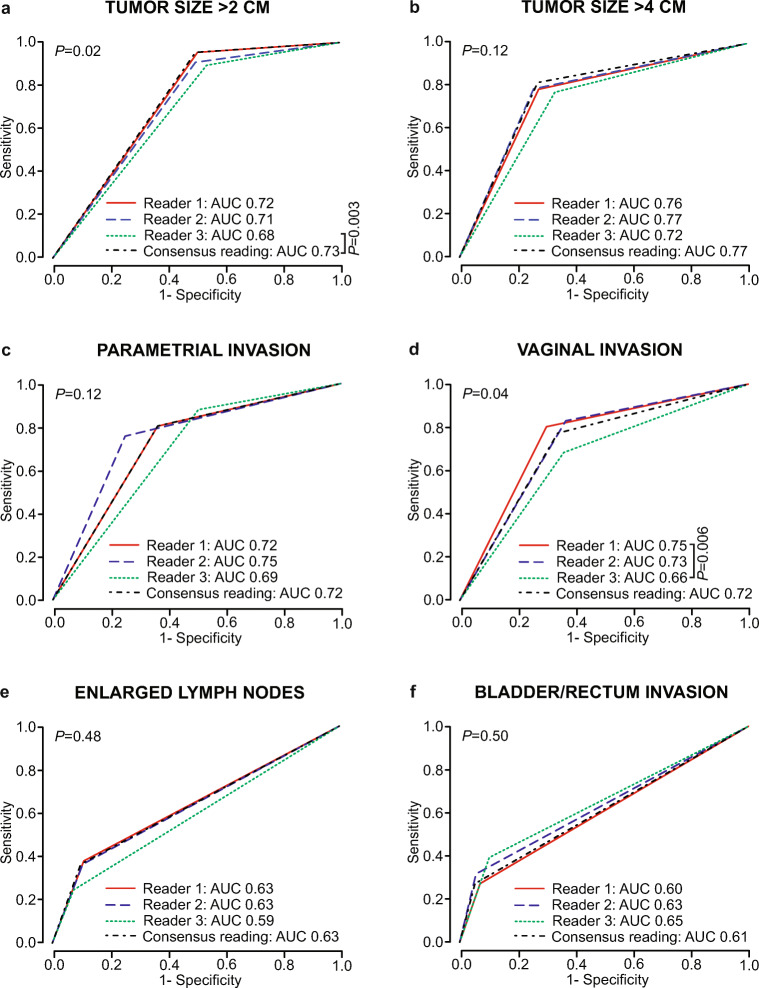
Time-dependent receiver operating characteristic (ROC) curves for prediction of disease-specific death at 5 years after primary diagnosis for MRI–derived tumor size > 2 cm (**a**), tumor size > 4 cm (**b**), parametrial invasion (**c**), vaginal invasion (**d**), enlarged lymph nodes (defined as pelvic/paraaortic lymph nodes with short-axis diameter > 1 cm) (**e**), and bladder/rectum invasion (**f**), for the three readers and the consensus reading. *p* values refer to the test of equal AUC values across readers and consensus reading. For the pairwise comparisons, only significant *p* values are given (after Holm–Bonferroni correction: *p* < 0.008)

For predicting disease-specific death, the ROC curves for tumor size > 2 cm, tumor size > 4 cm, parametrial invasion, vaginal invasion, enlarged lymph nodes, and bladder/rectum invasion yielded predominantly similar AUCs across readers/consensus reading (Fig. 3). However, for tumor size > 2 cm and vaginal invasion, reader 3 had significantly lower AUCs than consensus reading/reader 1 (*p* = 0.003 and *p* = 0.006, respectively) (Fig. 3).

### Imaging parameters and prediction of survival

**Fig. 4:**
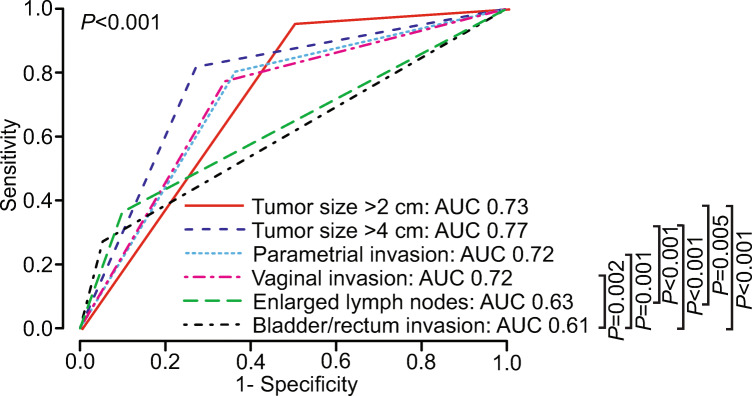
Time-dependent ROC curves for prediction of disease-specific death at 5 years after primary diagnosis for MRI–derived tumor size > 2 cm, tumor size > 4 cm, parametrial invasion, vaginal invasion, enlarged lymph nodes (defined as pelvic/paraaortic lymph nodes with short-axis diameter > 1 cm), and bladder/rectum invasion (consensus reading for all variables). *p* values refer to the test of equal AUC values across the MRI–derived staging parameters. For the pairwise comparisons, only significant *p* values are given (after Holm–Bonferroni correction: *p* < 0.005)

Time-dependent ROC curves for predicting disease-specific death at 5 years for the different MRI–derived staging parameters (consensus reading) yielded AUCs ranging from 0.61 to 0.77 with highest value for tumor size > 4 cm (AUC = 0.77), followed by tumor size > 2 cm (AUC = 0.73), parametrial invasion (AUC = 0.72), and vaginal invasion (AUC = 0.72) (Fig. 4).

The MRI–derived staging parameters large tumor size (in three categories: ≤ 2 cm; > 2 and ≤ 4 cm; > 4 cm), parametrial invasion, vaginal invasion, enlarged lymph nodes suggestive of metastases, and bladder/rectum invasion were associated with reduced disease-specific survival (*p* < 0.001 for all) (Table 4). However, in a multivariable model including the same imaging variables, only tumor size and bladder/rectum invasion independently predicted poor survival, whereas only tumor size remained significant when adjusting for patient age, histologic type, and primary treatment received (Table 4). When grouping patients according to tumor size categories (≤ 2 cm; > 2 and ≤ 4 cm; > 4 cm), higher tumor size category yielded a stepwise reduction in disease-specific and progression-free survival (*p* ≤ 0.001 for all) (Fig. 2b and Suppl. Figure 1b).

**Table 4:** Cox regression analysis of MRI–derived 2018 FIGO staging parameters (consensus reading) and clinicopathological patient characteristics for prediction of disease-specific survival in 416 patients with cervical cancer

	Univariable HR (95% CI)		*p*	Multivariable^a^ HR (95% CI)		*p*	Multivarible^**b**^ HR (95% CI)		*p*
Imaging variables (*n* = 416)									
Tumor size			**< 0.001**			**< 0.001**			**< 0.001**
≤ 2 cm	1.0			1.0			1.0		
>2 and ≤ 4 cm	5.3	(1.9–15.0)	**0.001**	5.7	(1.9–17.4)	**0.002**	2.7	(0.9–8.5)	0.08
> 4 cm	18.7	(7.5–46.6)	**< 0.001**	16.6	(5.1–54.1)	**< 0.001**	8.6	(2.8–26.4)	**< 0.001**
Parametrial invasion	5.6	(3.3–9.5)	**< 0.001**	0.8	(0.4–1.6)	0.49			
Vaginal invasion	4.8	(2.9–8.0)	**< 0.001**	1.0	(0.6–1.9)	0.92			
Enlarged lymph nodes^c^	3.9	(2.5–6.3)	**< 0.001**	1.5	(0.9–2.5)	0.12			
Bladder/rectum invasion	5.3	(3.2–8.8)	**< 0.001**	2.0	(1.2–3.4)	**0.01**	1.6	(0.9–2.8)	0.10
Clinicopathological variables (*n* = 416)									
Age at primary diagnosis, per decade	1.7	(1.5–1.9)	**< 0.001**				1.5	(1.2–1.7)	**< 0.001**
Histologic type			**0.003**						**0.005**
Squamous cell carcinoma	1.0						1.0		
Adenocarcinoma	0.8	(0.5–1.6)	0.50				1.6	(0.8–3.1)	0.16
Other^d^	2.7	(1.5–5.1)	**0.002**				3.0	(1.5–5.9)	**0.001**
Primary treatment			**< 0.001**						**< 0.001**
Surgery alone^e^	1.0						1.0		
Surgery and adjuvant therapy^f^	5.3	(2.2–12.8)	**< 0.001**				1.5	(0.5–4.1)	0.44
Radiotherapy ± chemotherapy alone	9.9	(4.9–20.2)	**< 0.001**				1.7	(0.7–4.2)	0.27
Palliative treatment	198.7	(72.2–546.5)	**< 0.001**				26.3	(7.6–91.6)	**< 0.001**

Significant *p* values are given in bold

*CI* confidence interval, *FIGO* International Federation of Gynecology and Obstetrics, *HR* hazard ratio

^a^Including all listed imaging variables

^b^Including the imaging variables tumor size and bladder/rectum invasion in addition to patient age at primary diagnosis, histologic tumor type, and primary treatment received

^c^Defined as pelvic/paraaortic lymph nodes with short-axis diameter > 1 cm

^d^Adenosquamous, neuroendocrine, and undifferentiated carcinomas

^e^Conization, trachelectomy, or hysterectomy ± bilateral salpingectomy/salpingo-oophorectomy

^f^Chemoradiation combined, chemotherapy only, or radiotherapy only

### MRI–derived assessments of tumor size > 4 cm and parametrial invasion refines prognostication

Patients with MRI–derived tumor size > 4 cm or parametrial invasion were more frequently diagnosed with squamous histology (Table 5). In patients with recordings on clinical tumor size (≤/> 4 cm) (*n* = 230), 75% (172/230) had the same tumor size category on MRI (Table 5). In 50 out of 155 (32%) patients with clinical tumor size ≤ 4 cm, MRI indicated tumor size > 4 cm, whereas in 8 out of 75 (11%) patients with clinical tumor size > 4 cm, MRI showed tumor size ≤ 4 cm. Incorporating MRI tumor size (≤/> 4 cm) information into the 2009 FIGO stage would have resulted in upstaging of 32% (50/155) and downstaging of 11% (8/75) of the patients (Table 5). Furthermore, patients with clinical tumor size ≤ 4 cm but MRI–based tumor size > 4 cm had lower disease-specific and progression-free survival than patients with both clinical- and MRI–derived tumor size ≤ 4 cm (*p* < 0.001) (Fig. 2c and Suppl. Figure 1c).

**Table 5:** Clinicopathological characteristics in 416 patients with cervical cancer with MRI–derived tumor size ≤ 4 cm/> 4 cm and MRI indicating/not indicating parametrial invasion (from consensus reading)

	MRI–derived tumor size ≤ 4 cm (*n* = 268)	MRI–derived tumor size > 4 cm (*n* = 148)	*P*^a^	No parametrial invasion on MRI (*n* = 236)	Parametrial invasion on MRI (*n* = 180)	*P*^a^
Clinical tumor size (*n* = 230)			**< 0.001**			**< 0.001**
≤ 4 cm (*n* = 155)	105 (93%)	50 (43%)		80 (91%)	75 (53%)	
> 4 cm (*n* = 75)	8 (7%)	67 (57%)		8 (9%)	67 (47%)	
2009 FIGO stage (*n* = 416)						**< 0.001**
I–IIA (*n* = 296)				226 (96%)	70 (39%)	
IIB–IV (*n* = 120)				10 (4%)	110 (61%)	
Histologic type (*n* = 416)			**0.007**			**0.004**
Squamous cell carcinoma (*n* = 292)	177 (66%)	115 (78%)		152 (64%)	140 (78%)	
Adenocarcinoma (*n* = 92)	72 (27%)	20 (14%)		66 (28%)	26 (14%)	
Other^b^ (*n* = 32)	19 (7%)	13 (9%)		18 (8%)	14 (8%)	
Histologic grade (*n* = 343)			**0.004**			0.10
1 & 2 (*n* = 253)	164 (80%)	89 (65%)		140 (78%)	113 (69%)	
3 (*n* = 90)	42 (20%)	48 (35%)		40 (22%)	50 (31%)	

Significant *p* values are given in bold

*FIGO* International Federation of Gynecology and Obstetrics

^a^Chi-square test

^b^Adenosquamous, neuroendocrine, and undifferentiated carcinomas

Parametrial invasion on MRI was diagnosed in 24% (70/296) of patients with 2009 FIGO I–IIA (clinically staged without parametrial invasion) (Table 5), and these patients had reduced disease-specific and progression-free survival (*p* = 0.008 and *p* = 0.007, respectively) (Fig. 2d and Suppl. Figure 1d). Incorporating MRI–assessed parametrial invasion into the 2009 FIGO would have resulted in upstaging of 24% (70/296) of the patients (Table 5).

## Discussion

Since 2018, staging information from diagnostic imaging has been formally incorporated in the FIGO system for cervical cancer, and routinely guides choice of treatment. We observed substantial interobserver agreement for most MRI–derived staging parameters, supporting the robustness of MRI staging in the 2018 FIGO system. Large MRI–measured tumor size, using the 2018 FIGO size categories, was associated with a stepwise reduction in disease-specific and progression-free survival, confirming the strong prognostic impact of tumor size in cervical cancer. Furthermore, MRI–assessed tumor size > 4 cm and parametrial invasion were associated with aggressive clinicopathological features and enabled improved risk stratification when compared to clinical assessments alone. Thus, this study demonstrates that the substantial interobserver agreement of MRI at primary diagnostic work-up in cervical cancer can translate into better prognostication, which is promising for the role of MRI in treatment tailoring.

Subjectivity in image interpretation may lead to variability that affects overall test reproducibility [30], and the interobserver agreement for important imaging findings is critical for the validity of an imaging method [31]. To our knowledge, this is the largest and most comprehensive study on interobserver agreement for pelvic MRI staging parameters in cervical cancer to date. Interestingly, maximum tumor size was the parameter yielding the highest interobserver agreement (overall *κ* = 0.76–0.80 for different size categories), being higher than that reported (*κ* = 0.46) in a previous smaller (*n* = 152) MRI study [21]. For parametrial invasion, vaginal invasion, and enlarged lymph nodes, we also identified substantial interobserver agreement (overall *κ* = 0.63, *κ* = 0.61, and *κ* = 0.63, respectively), being within the wide range of that previously reported (*κ* = 0.36–90) [19, 22–24].

Of notice, former studies assessing interobserver reproducibility for MRI–based cervical cancer staging parameters have used surgicopathological findings as reference standard, thus only including patients eligible for curative surgery based on clinical assessments [19, 21–24]. Hence, the lower prevalence of positive staging parameters for advanced FIGO stages in these studies makes these interobserver agreement metrics not necessarily comparable with that of the present study. Nevertheless, it seems reasonable to conclude that the interobserver agreement for central MRI staging parameters in this study is within the higher range of that previously reported. Importantly, clinical staging by pelvic examination under anesthesia reportedly yields lower agreement for assessing tumor size (*κ* = 0.42), parametrial invasion (*κ* = 0.31–0.43), and vaginal invasion (*κ* = 0.47–0.57) [32], thus indicating that MRI staging is more reproducible than clinical staging.

The inclusion of nodal status in the 2018 FIGO update reflects the importance of lymph node metastases as a pivotal prognostic factor and determinant of treatment algorithm in cervical cancer [6, 15, 33]. Notably, in the present study, MRI–assessed enlarged lymph nodes suggestive of metastases predicted reduced disease-specific survival in univariable analysis (*p* < 0.001), however, only tended to the same in multivariable analysis (*p* = 0.12). MRI has known limitations in diagnostic accuracy for diagnosing lymph node metastases and is reportedly being surpassed by FDG-PET/CT [34]. Limitations in accuracy of MRI may be due to the size criterion employed for pathologic lymph nodes, thus by definition missing the smaller lymph node metastases, and to challenges in distinguishing metastatic enlarged nodes from hyperplastic enlarged nodes [14, 35]. Thus, the lack of an independent prognostic impact of enlarged lymph nodes in this study may be explained by limited accuracy of MRI for lymph node staging. Importantly, although pathology is regarded as the reference standard for diagnosing lymph node metastases, the FIGO 2018 system allows the use of imaging, as it is non-invasive and easier to perform than surgical lymph node sampling [14, 25].

The updated 2018 FIGO stage IB comprises three subgroups (IB1–3) for tumor size ≤ 2 cm, > 2 and ≤ 4 cm, and > 4 cm, respectively [25]. Interestingly, we found that a higher tumor size category was linked to a stepwise reduction in disease-specific survival (*p* ≤ 0.001 for all) and that tumor size > 4 cm yielded the highest AUC (AUC = 0.77) among all MRI staging parameters for the prediction of disease-specific death. These findings are consistent with the growing body of literature uniformly reporting a strong association between large tumor size and poor prognosis in cervical cancer [6, 8–13].

Notably, tumor size > 4 cm and parametrial invasion at MRI were associated with aggressive clinicopathological features, and patients clinically staged as negative for these findings but with positive MRI findings had significantly reduced survival. Furthermore, incorporating MRI–derived information on tumor size (≤/> 4 cm) and parametrial invasion to the clinical 2009 FIGO staging would have resulted in an upstaging of 32% (50/155) and 24% (70/296) of the patients, respectively. Importantly, the evaluation of tumor size [20] and parametrial invasion [16] by MRI reportedly yields higher agreement with pathology than that of clinical assessment in cervical cancer, supporting that staging by MRI produces more accurate stage designation than clinical staging.

This study has some limitations. First, the MRI examinations were performed during 2002–2017 using various scanners and protocols, which may have affected our results. However, the demonstrated robustness of MRI for staging and prognostication despite these technical variations makes it more likely that our findings are generalizable, and that this study set-up more accurately mimics the value of MRI in a standard setting. Second, the study of interobserver reliability could have been more extensive and ideally included more readers with variable levels of expertise from different institutions. Third, we did not assess intraobserver variability, which is normally lower than the interobserver variability. Lastly, since a large proportion of the patients did not undergo surgery, our study is based on the assessment of agreement without surgicopathological reference standard, hence not indicative of diagnostic accuracy.

In summary, substantial interobserver agreement of MRI–based 2018 FIGO staging parameters supports the robustness of MRI staging in the 2018 FIGO system. Furthermore, the inclusion of MRI staging parameters into stage assignment yields refined risk stratification compared with former clinical 2009 FIGO staging. This study thus demonstrates the potential impact of MRI enabling better prognostication and treatment tailoring in cervical cancer.

## Supplementary information

ESM 1 (DOCX 415 kb)

## Abbreviations

AUC: Area under the curve
CT: Computed tomography
DWI: Diffusion-weighted imaging
FDG-PET/CT: ^18^F-fluorodeoxyglucose positron emission tomography/computed tomography
FIGO: International Federation of Gynecology and Obstetrics
IQR: Interquartile range
MRI: Magnetic resonance imaging
ROC: Receiver operating characteristic
T1W: T1-weighted
T2W: T2-weighted

## Declarations

### Guarantor

The scientific guarantor of this publication is Professor Ingfrid S. Haldorsen.

### Statistics and Biometry

Øyvind Salvesen (one of the co-authors) is an expert in medical statistics.

### Study subjects or cohorts overlap

The presented study subjects are all included in the biobank for cervical cancer and clinical database at our institution, and overlapping cases have been presented in previous publications primarily focusing on clinicopathologic and molecular/genetic markers in cervical cancer. One previous small study (n = 53) have presented PET-CT parameters for overlapping cases included in this MRI study but with an entirely different aim and no overlapping results.

Halle MK, Sødal M, Forsse D et al (2021) A 10-gene prognostic signature points to LIMCH1 and HLA-DQB1 as important players in aggressive cervical cancer disease. Br J Cancer. 10.1038/s41416-021-01305-0

Halle MK, Ojesina AI, Engerud H et al (2017) Clinicopathologic and molecular markers in cervical carcinoma: a prospective cohort study. Am J Obstet Gynecol 217:432.e431-432.e417

Halle MK, Krakstad C, Engerud H, Bertelsen B, Salvesen HB (2014) Molecular profiling in fresh tissue with high tumor cell content promotes enrichment for aggressive adenocarcinomas in cervix. Pathol Res Pract 210:774-778

Bollineni VR, Ytre-Hauge S, Gulati A et al (2018) The prognostic value of preoperative FDG-PET/CT metabolic parameters in cervical cancer patients. European Journal of Hybrid Imaging 2:24

### Methodology

- retrospective
- diagnostic or prognostic study
- performed at one institution
