# Supplementary material for: Interobserver agreement and prognostic impact for MRI–based 2018 FIGO staging parameters in uterine cervical cancer
Source: Eur Radiol. 2022 Mar 24;32(9):6444–55. doi: 10.1007/s00330-022-08666-x (PMC9381622; doi:10.1007/s00330-022-08666-x)

| **Supplementary Table 1**. Acquisition parameters for a subset of 123 of the total 416 pelvic MRI examinations included in this study. The selected subset is considered illustrative of the entire study cohort. Values are given as median (range) | | | | | | | | | | | | | |
| --- | --- | --- | --- | --- | --- | --- | --- | --- | --- | --- | --- | --- | --- |
| MRI scanner | *n* | Sequence | Plane | TR,  ms | TE,  ms | FA, degree | FOV,  mm^2^ | Matrix shape | Slice thickness, mm | Interslice gap,  mm | Number of slices | Pixel size,  mm^2^ | NEX |
| GE Healthcare 1.5T | 9 |  |  |  |  |  |  |  |  |  |  |  |  |
|  |  | T2 TSE | Ax/  AxObl | 3158  (1846–3352) | 81  (79–96) | 160  (160–160) | 180x180  (180x180–200x200) | 512x512  (512x512–512x512) | 3.0  (3.0–3.0) | 0  (0–0) | 30  (17–32) | 0.4x0.4  (0.4x0.4–0.4x0.4) | 2  (2–2) |
|  |  | DWI | Ax/  AxObl | 4000  (3000–6707) | 52  (51–74) | 90  (90–90) | 350x350  (300x300–400x400) | 256x256  (256x256–256x256) | 5.0  (4.0–8.0) | 0.5  (0.4–2.0) | 25  (17–40) | 1.4x1.4  (1.2x1.2–1.6x1.6) | 2  (1–2) |
| Siemens Healthineers 1.5T | 51 |  |  |  |  |  |  |  |  |  |  |  |  |
|  |  | T2 TSE | Ax/  AxObl | 4790  (3000–7075) | 100  (83–114) | 150  (150–180) | 180x180  (160x160–200x200) | 512x512  (256x256–512x512) | 3.0  (3.0–5.0) | 0.5  (0–0.8) | 25  (15–37) | 0.4x0.4  (0.3x0.3–0.8x0.8) | 2  (1–3) |
|  |  | DWI | Ax/  AxObl | 3200  (2600–5400) | 82  (60–85) | 90  (90–90) | 250x250  (250x225–285x380) | 144x144  (128x128–256x256) | 4.0  (3.0–6.0) | 0.6  (0.4–1.5) | 22  (20–30) | 1.6x1.6  (1.0x1.0–2.3x2.3) | 10  (4–12) |
| Siemens Healthineers 3T | 27 |  |  |  |  |  |  |  |  |  |  |  |  |
|  |  | T2 TSE | Ax/  AxObl | 4610  (4000–5780) | 94  (89–97) | 148  (120–160) | 200x200  (200x200–300x300) | 384x384  (320x320–384x384) | 3.0  (3.0–5.0) | 0.3  (0–2.0) | 24  (24–35) | 0.5x0.5  (0.5x0.5–0.8x0.8) | 2  (2–3) |
|  |  | DWI | Ax/  AxObl | 5640  (4000–7600) | 63  (54–78) | 180  (90–180) | 200x200  (200x200–360x360) | 144x144  (100x100–160x160) | 3.0  (3.0–5.0) | 0.4  (0.3–3.0) | 25  (24–30) | 1.4x1.4  (1.3x1.3–2.5x2.5) | 2  (1–12) |
| Philips Healthcare 1.5T | 27 |  |  |  |  |  |  |  |  |  |  |  |  |
|  |  | T2 TSE | Ax/  AxObl | 5362  (2482–5362) | 100  (80–125) | 90  (90–90) | 205x205  (180x180–215x215) | 512x512  (256x256–528x528) | 3.0  (2.5–5.0) | 0.3  (0–0.5) | 26  (20–40) | 0.4x0.4  (0.3x0.3–0.8x0.8) | 6  (1–8) |
|  |  | DWI | Ax/  AxObl | 1716  (1300–5422) | 69  (64–84) | 90  (90–90) | 375x375  (160x160–410x410) | 256x256  (128x128–288x288) | 5.0  (4.0–7.0) | 1.0  (0.3–1.0) | 30  (14–35) | 1.5x1.5  (1.3x1.3–2.0x2x0) | 3  (3–6) |
| Philips Healthcare 3T | 9 |  |  |  |  |  |  |  |  |  |  |  |  |
|  |  | T2 TSE | Ax/  AxObl | 4075  (3955–4427) | 110  (110–110) | 90  (90–90) | 180x180  (180x180–180x180) | 512x512  (512x512–512x512) | 2.5  (2.5–2.5) | 0.3  (0.3–0.3) | 35  (30–35) | 0.4x0.4  (0.4x0.4–0.4x0.4) | 2  (2–2) |
|  |  | DWI | Ax/  AxObl | 3280  (3256–3280) | 85  (84–85) | 90  (90–90) | 280x280  (280x280–280x280) | 352x352  (352x352–352x352) | 4.0  (4.0–4.0) | 0.4  (0.4–0.4) | 33  (33–33) | 0.8x0.8  (0.8x0.8–0.8x0.8) | 2  (2–2) |
| *n* refers to number of patients  Ax, axial; AxObl, axial oblique; DWI, diffusion weighted imaging; FA, flip angle; FOV, field of view; TE, time to echo; TR, repetition time; TSE, turbo spin echo; NEX, number of exitations | | | | | | | | | | | | | |

| **Supplementary Table 2** Primary treatment given to 416 patients with cervical cancer according to 2009 FIGO stage | | | | | |
| --- | --- | --- | --- | --- | --- |
|  | FIGO stage I  *n* = 282 | FIGO stage II  *n* = 80 | FIGO stage III  *n* = 37 | FIGO stage IV  *n* = 17 | Total *n* |
| **Primary treatment** |  |  |  |  |  |
| Surgery alone^a^ | 208 (74%) | 2 (3%) | 0 (0%) | 0 (0%) | 210 (51%) |
| Surgery and  adjuvant therapy^b^ | 44 (16%) | 5 (6%) | 0 (0%) | 2 (12%) | 51 (12%) |
| Radiotherapy −/+ chemotherapy alone | 30 (11%) | 72 (90) | 34 (92%) | 11 (65%) | 147 (35%) |
| Palliative treatment | 0 (0%) | 1 (1%) | 3 (8%) | 4 (24%) | 8 (2%) |
| FIGO, International Federation of Gynecology and Obstetrics.  ^a^Conization, trachelectomy, or hysterectomy −/+ bilateral salpingectomy/salpingo-oophorectomy. ^b^Chemoradiation combined, chemotherapy only, or radiotherapy only. | | | | | |

| **Supplementary Table 3** Positive MRI findings in the subgroups of patients with visible tumor | | | | |
| --- | --- | --- | --- | --- |
|  | Reader 1  *n* (%) | Reader 2  *n* (%) | Reader 3  *n* (%) | Consensus reading  *n* (%) |
| Visible tumor | *n* = 276 | *n* = 273 | *n* = 259 | *n* = 270 |
|  |  |  |  |  |
| Tumor size >2 cm | 235 (85) | 234 (86) | 242 (93) | 235 (87) |
| Tumor size >4 cm | 149 (54) | 142 (52) | 166 (64) | 148 (55) |
| Tumor size, three categories |  |  |  |  |
| ≤2 cm | 41 (15) | 39 (14) | 17 (7) | 35 (13) |
| >2 and ≤4 cm | 86 (31) | 92 (34) | 76 (29) | 87 (32) |
| >4 cm | 149 (54) | 142 (52) | 166 (64) | 148 (55) |
| Parametrial invasion | 180 (65) | 144 (53) | 230 (89) | 180 (67) |
| Vaginal invasion | 161 (58) | 186 (68) | 168 (65) | 173 (64) |
| Limited to upper two-thirds | 134 (49) | 176 (64) | 111 (43) | 153 (57) |
| Extension to lower one-third | 27 (10) | 10 (4) | 57 (23) | 20 (7) |
| Pelvic-sidewall invasion | 1 (0) | 10 (4) | 5 (2) | 3 (1) |
| Hydroureter | 5 (2) | 6 (2) | 1 (0) | 3 (1) |
| Enlarged lymph nodes^a^ | 61 (22) | 61 (22) | 42 (16) | 57 (21) |
| Bladder/rectum invasion | 45 (16) | 41 (15) | 59 (23) | 36 (13) |
| ^a^Defined as pelvic/paraaortic lymph nodes with short axis diameter >1 cm. | | | | |

**Supplementary Figure 1** Kaplan–Meier survival curves depicting significantly reduced progression-free survival in patients with:

**a** 2009 FIGO stages IB2–IIA and ≥IIB compared to stages ≤IB1,

**b** Higher MRI-derived tumor size categories,

**c** Clinical tumor size ≤4 cm but MRI-derived tumor size >4 cm,

**d** 2009 FIGO stages I–IIA but parametrial invasion at MRI.

Progression was defined as local recurrence/progression in the pelvis or new metastases in the abdomen or at distant sites, confirmed by biopsy or by imaging (CT, MRI, and/or FDG-PET/CT).

For each category: total number of cases/number of cases with progression.

FIGO, International Federation of Gynecology and Obstetrics.


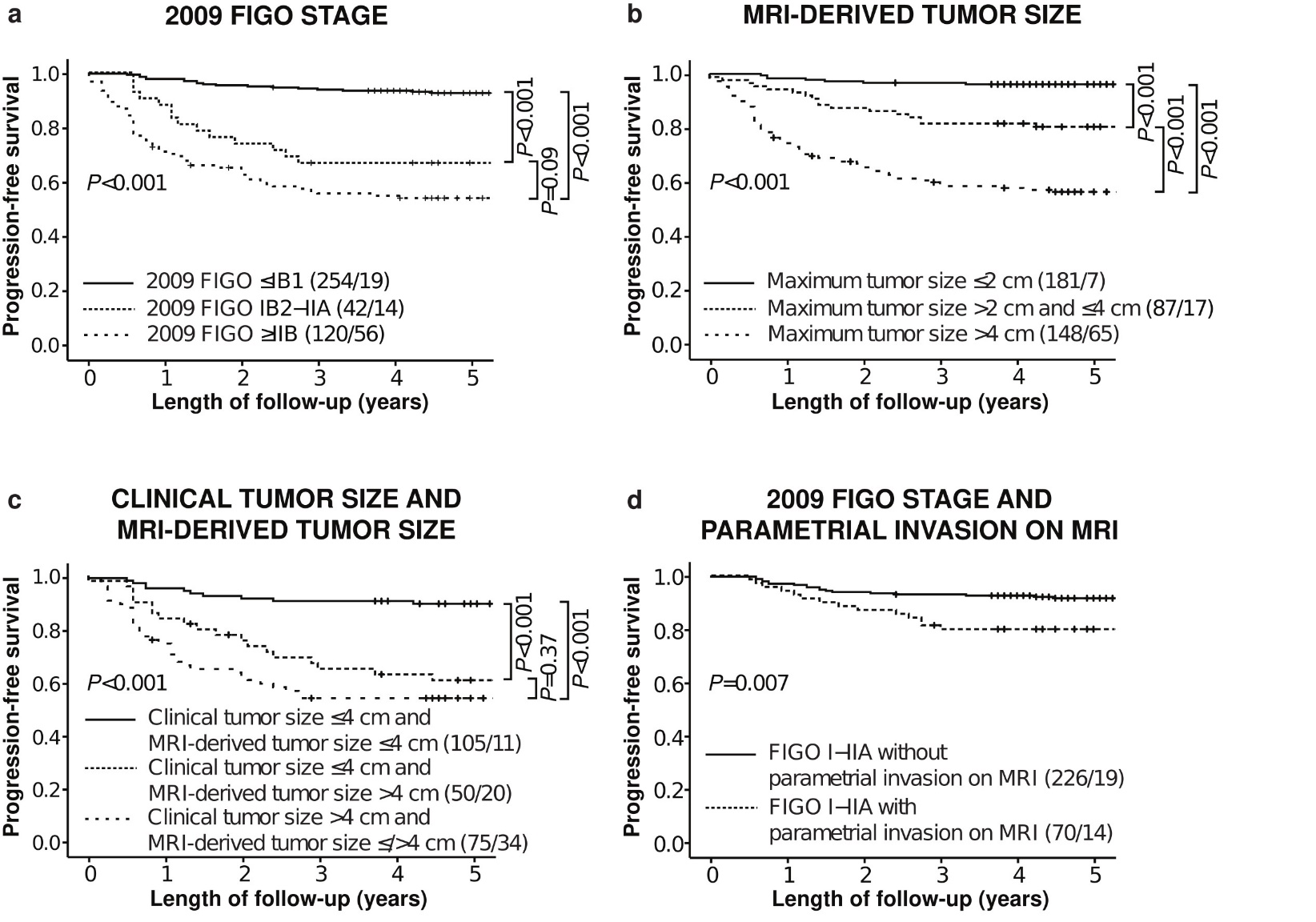

Supplement: Supplementary file 1 — (DOCX 415 kb) [file 330_2022_8666_MOESM1_ESM.docx]
